# SARS-CoV-2 Bearing a Mutation at the S1/S2 Cleavage Site Exhibits Attenuated Virulence and Confers Protective Immunity

**DOI:** 10.1128/mBio.01415-21

**Published:** 2021-08-24

**Authors:** Michihito Sasaki, Shinsuke Toba, Yukari Itakura, Herman M. Chambaro, Mai Kishimoto, Koshiro Tabata, Kittiya Intaruck, Kentaro Uemura, Takao Sanaki, Akihiko Sato, William W. Hall, Yasuko Orba, Hirofumi Sawa

**Affiliations:** a Division of Molecular Pathobiology, International Institute for Zoonosis Control, Hokkaido University, Sapporo, Japan; b Shionogi & Co., Ltd., Osaka, Japan; c Laboratory of Biomolecular Science, Faculty of Pharmaceutical Science, Hokkaido University, Sapporo, Japan; d International Collaboration Unit, International Institute for Zoonosis Control, Hokkaido University, Sapporo, Japan; e National Virus Reference Laboratory, School of Medicine, University College of Dublin, Ireland; f Global Virus Network, Baltimore, Maryland, USA; g One Health Research Center, Hokkaido University, Sapporo, Japan; Virginia Tech; Virginia Polytechnic Institute and State University

**Keywords:** furin cleavage site, SARS-CoV-2, spike, attenuation, neutralizing antibodies

## Abstract

Severe acute respiratory syndrome-coronavirus-2 (SARS-CoV-2) possesses a discriminative polybasic cleavage motif in its spike protein that is recognized by the host furin protease. Proteolytic cleavage activates the spike protein, thereby affecting both the cellular entry pathway and cell tropism of SARS-CoV-2. Here, we investigated the impact of the furin cleavage site on viral growth and pathogenesis using a hamster animal model infected with SARS-CoV-2 variants bearing mutations at the furin cleavage site (S gene mutants). In the airway tissues of hamsters, the S gene mutants exhibited low growth properties. In contrast to parental pathogenic SARS-CoV-2, hamsters infected with the S gene mutants showed no body weight loss and only a mild inflammatory response, thereby indicating the attenuated variant nature of S gene mutants. This transient infection was sufficient for inducing protective neutralizing antibodies that cross-react with different SARS-CoV-2 lineages. Consequently, hamsters inoculated with S gene mutants showed resistance to subsequent infection with both the parental strain and the currently emerging SARS-CoV-2 variants belonging to lineages B.1.1.7 and P.1. Taken together, our findings revealed that the loss of the furin cleavage site causes attenuation in the airway tissues of hamsters and highlighted the potential benefits of S gene mutants as potential immunogens.

## INTRODUCTION

In humans, severe acute respiratory syndrome-coronavirus-2 (SARS-CoV-2) causes an infectious respiratory disease—novel coronavirus disease 2019 (COVID-19). Patients with severe COVID-19 pneumonia exhibit high expression levels of proinflammatory cytokines leading to hyper-inflammation with tissue damage. In particular, interleukin 6 (IL-6) plays a pivotal role in the hyper-inflammatory response during the acute phase of viral infection and is associated with disease severity (1, 2). During the global spread of SARS-CoV-2, variants carrying adaptive mutations in their spike gene have been identified in different countries, raising global concerns regarding disease severity, transmissibility, and immune escape against the ancestral SARS-CoV-2 (3–5).

Syrian hamsters and nonhuman primates are highly susceptible to SARS-CoV-2 infection and develop pneumonia with profound inflammatory responses (6–10). Moreover, transgenic mice expressing human angiotensin-converting enzyme 2 (ACE2) and mouse transduced human-ACE2 have been used to investigate SARS-CoV-2 infection; however, owing to the inaccessibility of mouse ACE2 (11–13), laboratory mice are resistant to infection with some clinical SARS-CoV-2 strains. These animals recover from the transient infection and acquire protective neutralizing antibodies (10, 11). To date, hamsters are widely used as an animal model for conducting research on pathogenicity and host immune responses as well as for the development of vaccines and antiviral drugs (14, 15).

The spike (S) protein of SARS-CoV-2 is a homotrimeric glycoprotein located on the virion surface; it plays a major role in virus entry into target cells by binding to specific entry receptors (16). The S protein possesses a discriminative polybasic cleavage motif at the S1/S2 boundary that is recognized by the host furin protease and is required for S protein cleavage into S1 and S2 subunits (13, 17–19). Importantly, this proteolytic cleavage affects the virus entry pathway (direct fusion or endocytosis) and cell tropism (17–19). However, our previous findings and those from other research groups suggested that SARS-CoV-2 variants bearing mutations at the furin cleavage site can be selected following passaging in Vero cells (18, 20–26). Although these mutants have been well characterized using cell-based assays, the role of the furin cleavage site in cell tropism and pathogenicity *in vivo* remains to be elucidated. Notably, the loss of the furin cleavage site results in the attenuation of pathogenicity of SARS-CoV-2 in hamsters and human-ACE2 transgenic mice (13, 20, 26).

In the present study, we characterized *in vivo* growth and pathogenicity of SARS-CoV-2 S gene mutants bearing deletions or substitutions at the furin cleavage sites of their S proteins (18) using a hamster model. We examined the attenuation and mild inflammatory response following infection with the S gene mutants using histopathological and cytokine expression analyses. Hamsters infected with the attenuated mutants developed neutralizing antibodies that cross-reacted with different lineages of SARS-CoV-2; therefore, we examined whether the primary infection with an S gene mutant could protect hamster recipients from both reinfection with the parental pathogenic SARS-CoV-2 and the currently emerging SARS-CoV-2 variants belonging to lineages B.1.1.7 and P.1.

## RESULTS

### Low growth properties of SARS-CoV-2 S gene mutants in Syrian hamsters.

**FIG 1:**
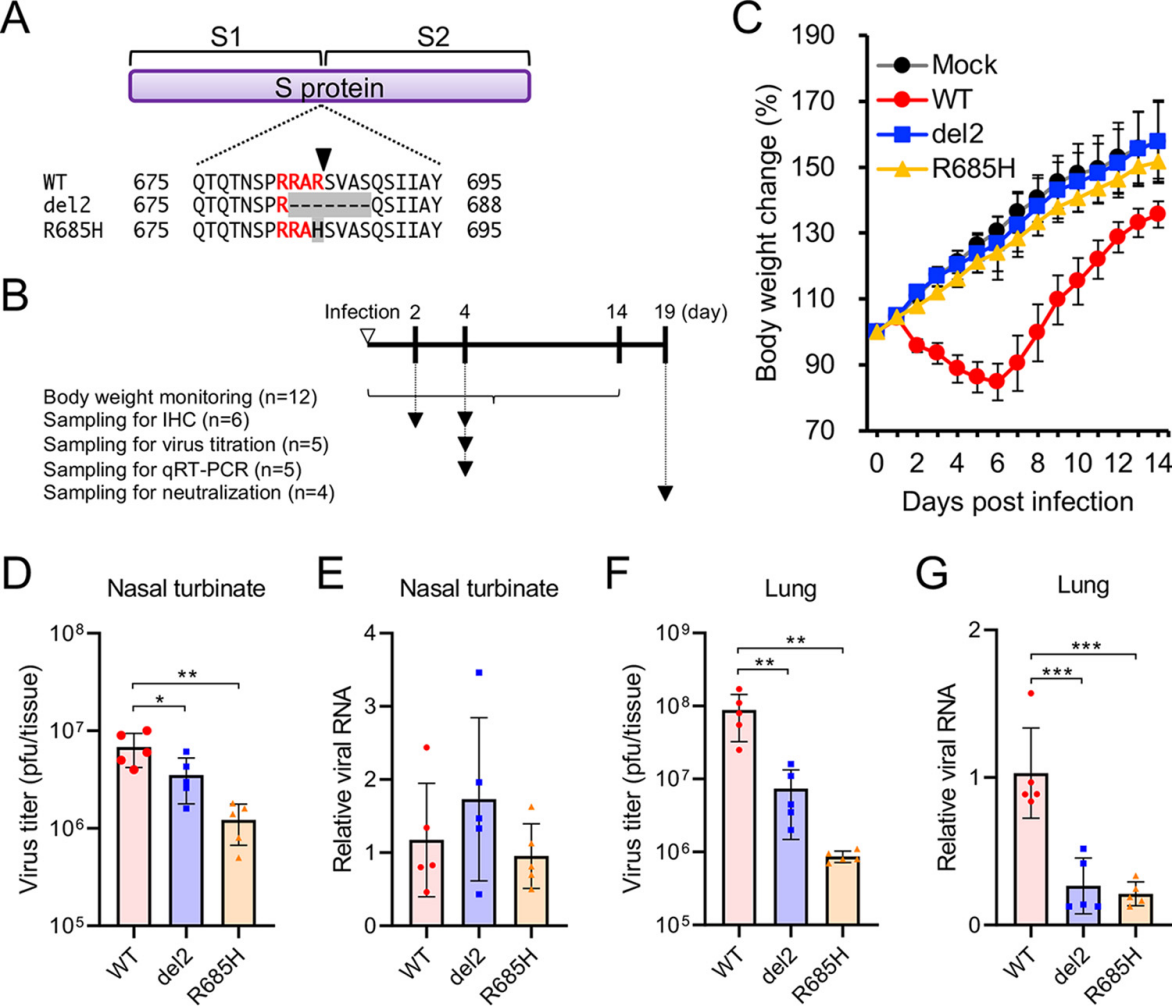
Growth of SARS-CoV-2 S gene mutants in Syrian hamsters. (A) Nascent full-length S protein is cleaved into S1 and S2 subunits at the S1/S2 cleavage site. Multiple amino acid sequence alignments were focused on the S1/S2 cleavage site of wild-type (WT) and S gene mutants (del2 and R685H). The arrowhead indicates the cleavage site. (B) Schematic of infection and sampling. Hamsters were intranasally infected with 1.5 × 10^4^ PFU of WT or S gene mutants. Body weight was monitored for 14 days. Tissues and serum were harvested at the indicated time points. The numbers of examined hamsters in each group are represented in the parentheses. (C) Syrian hamsters were infected with SARS-CoV-2 WT or S gene mutants (del2 and R685H) via the intranasal route. The mean of body weight changes of mock- or virus-infected hamsters (*n *=* *12 per group) was monitored daily. (D and F) Infectious titers in the nasal turbinate (D) and lung (F) of hamsters at 4 days postinfection (dpi). Viral titers in the cultures were determined using plaque assays. (E and G) Viral RNA levels relative to the WT virus in the nasal turbinate (E) and lung (G) of Syrian hamsters at 4 dpi. The viral RNA levels were quantified using qRT-PCR and normalized to β-actin expression levels. One-way analysis of variance with Tukey’s test was used to determine the statistical significance of the differences in virus titers between the WT and S gene mutants. *, *P < *0.05; **, *P < *0.01; ***, *P < *0.001.

Syrian hamsters experimentally infected with SARS-CoV-2 via the intranasal route typically lose body weight until 6 to 7 days postinfection (dpi) (7–10). To examine the susceptibility of infection by S gene mutants, we inoculated hamsters with a clinical SARS-CoV-2 isolate, WK-521 (wild-type, WT) or S gene mutants (del2 and R685H) (Fig. 1A) (18). The hamsters were monitored daily and sacrificed for tissue and serum collection (Fig. 1B). Hamsters infected with WT virus showed body weight loss at 2 to 6 dpi; however, infection with S gene mutants showed no impact on the hamster body weight (Fig. 1C). The viral load of SARS-CoV-2 in hamsters reportedly decreased at 5 to 7 dpi (7–10). Therefore, we harvested nasal turbinate and lung tissues at 4 dpi for the quantification of infectious SARS-CoV-2 and its RNA. In the nasal turbinate tissues, infectious virus titers of S gene mutants were 2- to 6-fold lower than those of the WT virus, whereas no difference was observed in viral RNA levels using quantitative reverse transcription-PCR (qRT-PCR) (Fig. 1D and E). In the lungs, a markedly more evident difference in growth properties was observed between WT and S gene mutants. S gene mutants produced 12- to 100-fold lower levels of infectious virus, and viral RNA levels of S gene mutants were significantly lower than those of the WT virus (Fig. 1F and G). No compensatory mutation was identified in the S gene of S gene mutants in the nasal turbinate and lung tissues at 4 dpi. These results suggested that the S gene mutants exert low pathogenicity in hamsters and possess low growth capacity in the respiratory tissues of hamsters.

### Histopathology and cytokine profiles in the lungs of hamsters infected with SARS-CoV-2 S gene mutants.

**FIG 2:**
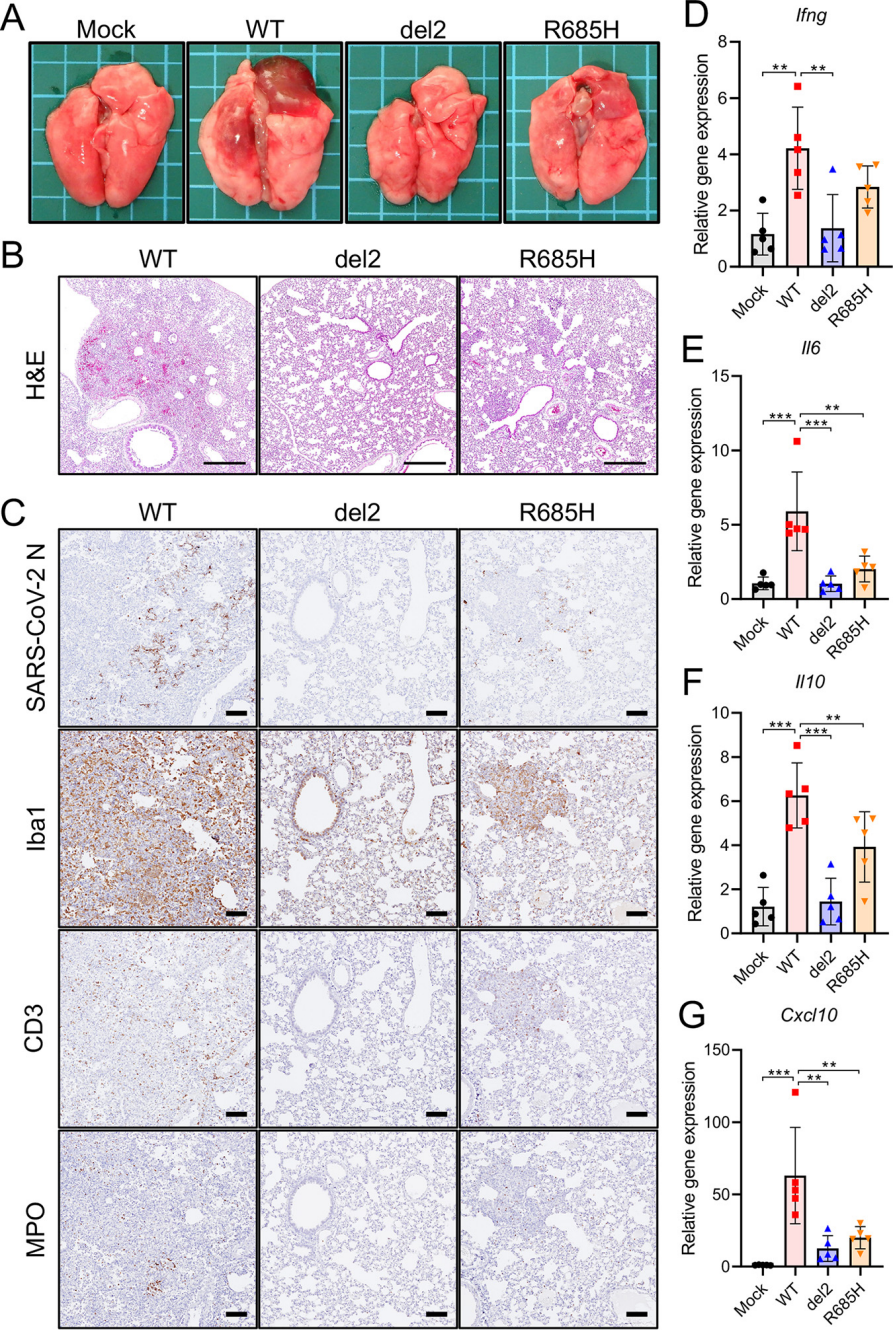
Pathological changes and immune response in the lung tissues of hamsters infected with SARS-CoV-2 S gene mutants. (A) Gross pathological images of the lungs of hamsters infected with WT or S gene mutants at 4 days postinfection (dpi). (B) Histopathological images of the lungs of hamsters infected with WT or S gene mutants at 4 dpi with H&E staining. Scale bars = 500 μm. (C) Immunohistochemistry for SARS-CoV-2 N protein, macrophage (Iba1), T cell (CD3), and neutrophil (MPO) markers. Cell nuclei were counterstained with hematoxylin. Scale bars = 100 μm. (D to G) Cytokine gene expression profile in lung tissues from hamsters at 4 dpi. Relative gene expression levels of the indicated cytokines in the lungs compared with those of lungs from mock-infected hamsters were examined using qRT-PCR. Data were normalized to β-actin. One-way analysis of variance with Tukey’s test was used to determine the statistical significance of the differences. **, *P < *0.01; ***, *P < *0.001.

We examined gross and histological changes in the lungs of hamsters inoculated with the SARS-CoV-2 S gene mutants. On gross examination, focal pulmonary consolidations and hyperemia were primarily observed in the hilar regions of hamsters infected with WT virus at 4 dpi (Fig. 2A). In contrast, in the lungs of hamsters infected with the S gene mutants, these gross pathological changes were limited or no apparent changes were noted (Fig. 2A). Immunohistochemistry identified viral antigens in the nasal, bronchial, and alveolar epithelia of hamsters at 2 dpi of both WT and S gene mutants (Fig. S1A and B). At 4 dpi, the histopathological examination conducted revealed pulmonary lesions with marked hemorrhage and inflammatory cell infiltration in the alveolar spaces of hamsters infected with the WT virus (Fig. 2B). Conversely, the histopathological changes of the lungs inoculated with the S gene mutants were relatively mild compared with those of the lungs inoculated with the WT virus. Immunohistochemistry showed widespread viral antigen-positive cells in the lung of hamsters infected with WT virus, which contrasted with the relatively limited distribution of viral antigen in the lungs infected with the S gene mutants (Fig. 2C). The inflammatory cells were composed primarily of ionized calcium-binding adaptor molecule 1 (Iba1)-positive macrophages (Fig. 2C), which are considered to induce severe immune damage; this finding was consistent with observations in severe COVID-19 cases (27, 28). Notably, we observed only limited inflammatory cell infiltration in the lungs infected with S gene mutants (Fig. 2C).

10.1128/mBio.01415-21.1 FIG S1 Viral antigen-positive cells in hamsters at 2 days postinfection. Immunohistochemistry of SARS-CoV-2 N in the nasal turbinate (A) and lung (B) tissues at 2 days postinfection (dpi) of SARS-CoV-2 WK-521 WT or S gene mutants. Cell nuclei were counterstained with hematoxylin. Scale bars = 100 μm. Download FIG S1, TIF file, 2.5 MB. Copyright © 2021 Sasaki et al. 2021 Sasaki et al. https://creativecommons.org/licenses/by/4.0/ This content is distributed under the terms of the Creative Commons Attribution 4.0 International license.

In accordance with human COVID-19, experimental infection with SARS-CoV-2 induced proinflammatory cytokine responses leading to extensive inflammatory cell infiltration in hamsters and mice (8, 12, 29). We examined the cytokine expression levels of the hamster lungs of WT and S gene mutants at 4 dpi using qRT-PCR. WT-infection significantly upregulated the expression of gamma interferon (IFN-γ), IL-6, IL-10, and CXCL10 (also known as IP-10) in the lungs compared with that in S gene mutants (Fig. 2D–2G). These results indicated that infection with S gene mutants resulted in an attenuated inflammatory response in the lungs of the hamsters.

### Low growth property of SARS-CoV-2 S gene mutants in primary human airway epithelium.

**FIG 3:**
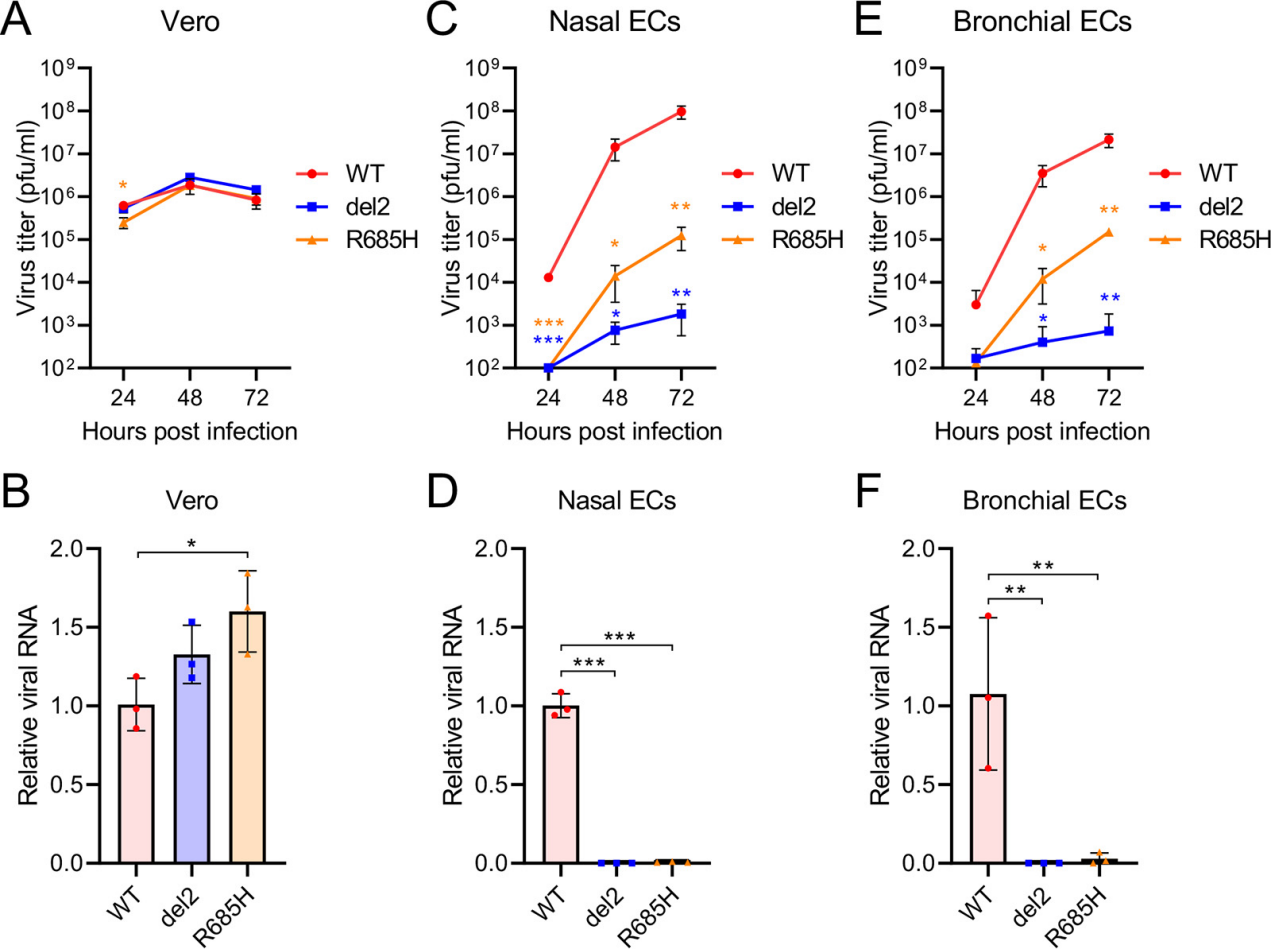
Growth of SARS-CoV-2 S gene mutants in *in vitro* cell culture. (A, C, and E) Growth curves of SARS-CoV-2 WT or S gene mutants in Vero cells (A), primary human nasal epithelial cells (C), and bronchial epithelial cells (E). Viral titers in the cultures were determined using a plaque assay. The values in the graphs are shown as the means ± SD of triplicates and are representative of two independent experiments with similar results. (B, D, and F) Viral RNA levels relative to WT virus in Vero cells (B), primary human nasal epithelial cells (D), and bronchial epithelial cells (F) at 48 h postinfection. The viral RNA levels were normalized to β-actin expression levels. One-way analysis of variance with Tukey’s test was used to determine the statistical significance of the differences between the WT and S gene mutants. *, *P < *0.05; **, *P < *0.01; ***, *P < *0.001.

We further evaluated the growth property of S gene mutants in human airway epithelium using three-dimensional (3D) reconstituted human nasal or bronchial epithelial cell models (nasal ECs or bronchial ECs, respectively) cultured at an air-liquid interface (30). In control Vero E6 cells, the progeny virus titers and viral RNA levels of S gene mutants were found to be equivalent to or higher than those of the WT virus (Fig. 3A and B). In contrast, the replication and growth of S gene mutants were impaired in human nasal ECs and bronchial ECs (Fig. 3C to F); this observation was consistent with the different growth properties of WT and S gene mutants in the respiratory airway of hamsters.

### Infection of SARS-CoV-2 S gene mutants induces protective neutralizing antibody.

**FIG 4:**
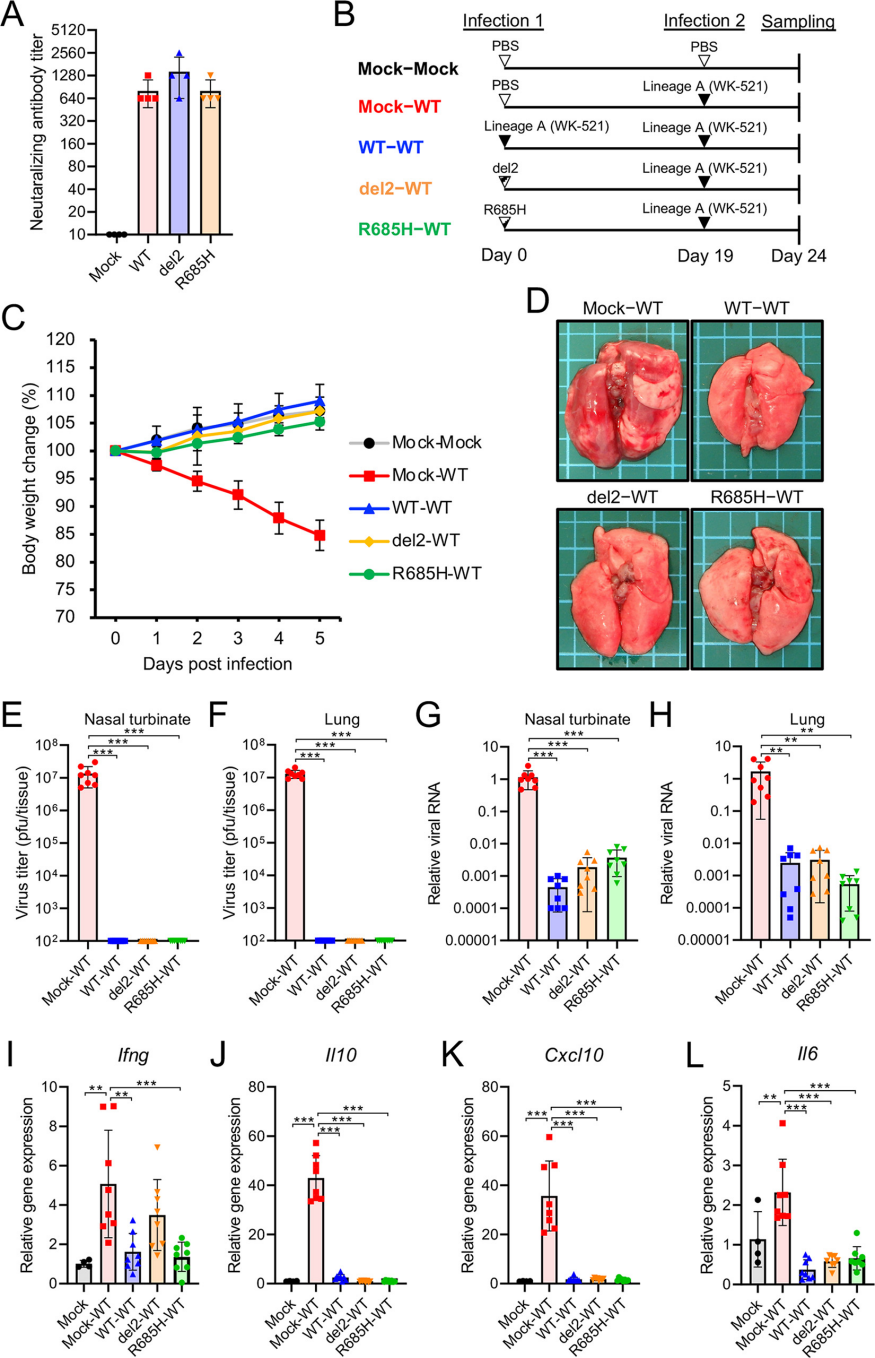
Reinfection of hamsters with SARS-CoV-2 WK-521 WT. (A) Neutralizing antibody titers in hamster serum at 19 dpi with WT or S gene mutants. (B) Schematic of primary infection, reinfection, and sampling. Hamsters were intranasally infected with 1.5 × 10^4^ PFU of WT or S gene mutants. At 19 days post-initial infection, hamsters were reinfected with 1.5 × 10^5^ PFU of WT virus. Mock-inoculated hamsters (mock-mock) and primary-infected hamsters (mock-WT) were used as controls. (C) Mean body weight changes of hamsters from 0 to 5 days postreinfection. Sample sizes were as follows: *n *=* *4 for the mock-mock group and *n *=* *8 for the remaining groups. (D) Gross pathological images of the lungs of hamsters at 5 days postreinfection. (E and F) Infectious virus titers in the nasal turbinate (E) and lung (F) tissues of hamsters at 5 days postreinfection. Viral titers in the cultures were determined using plaque assays. (G and H) Viral RNA levels relative to primary-infected hamsters (mock-WT) in the nasal turbinate (G) and lung (H) tissues of hamsters at 5 days postreinfection. The viral RNA levels were quantified using qRT-PCR and normalized to β-actin expression levels. (I to L) Relative gene expression levels of the indicated cytokines in the lungs compared with the levels in the lungs from mock-infected hamsters (mock-mock) were examined using qRT-PCR. Data were normalized to β-actin expression levels. One-way analysis of variance with Tukey’s test was used to determine the statistical significance of the differences. **, *P < *0.01; ***, *P < *0.001.

Individuals infected with SARS-CoV-2 typically present with detectable seroconversion at 10 to 14 dpi (31). Although the virus titers in the lungs of hamsters infected with S gene mutants were lower than those with the WT virus (Fig. 1D), both hamsters infected with either WT and those infected with the S gene mutants developed similar levels of neutralizing antibody titers at 19 dpi (Fig. 4A). To investigate the protective effect of the neutralizing antibodies, we rechallenged hamsters infected with either WT or S gene mutants with the WT virus (Fig. 4B). Hamsters inoculated with WT or S gene mutants at primary infection showed no body weight loss and no macroscopic changes in the lungs following the rechallenge with WT (WT-WT, del2-WT, and R685H-WT in Fig. 4C and D). In contrast, control hamsters inoculated with phosphate-buffered saline (PBS) at the primary infection point showed marked body weight loss and macroscopic changes in the lung following secondary infection with WT (mock-WT in Fig. 4C and D). Primary infection with WT and S gene mutants prevented the proliferation of rechallenged virus and decreased viral RNA levels in the nasal turbinate and lung tissues at 5 days postreinfection (Fig. 4E to H). Consistent with the inhibition of virus growth, cytokine levels in WT-WT-, del2-WT-, and R685H-WT-infected hamsters were significantly lower than those in Mock-WT-infected hamsters (Fig. 4I to L). These results indicated that infection with the attenuated S gene mutants induced protective neutralizing antibodies and reduced disease burden during reinfection with the WT virus.

### Cross-reactive antibody responses to SARS-CoV-2 variants B.1.1.7 and P.1.

**FIG 5:**
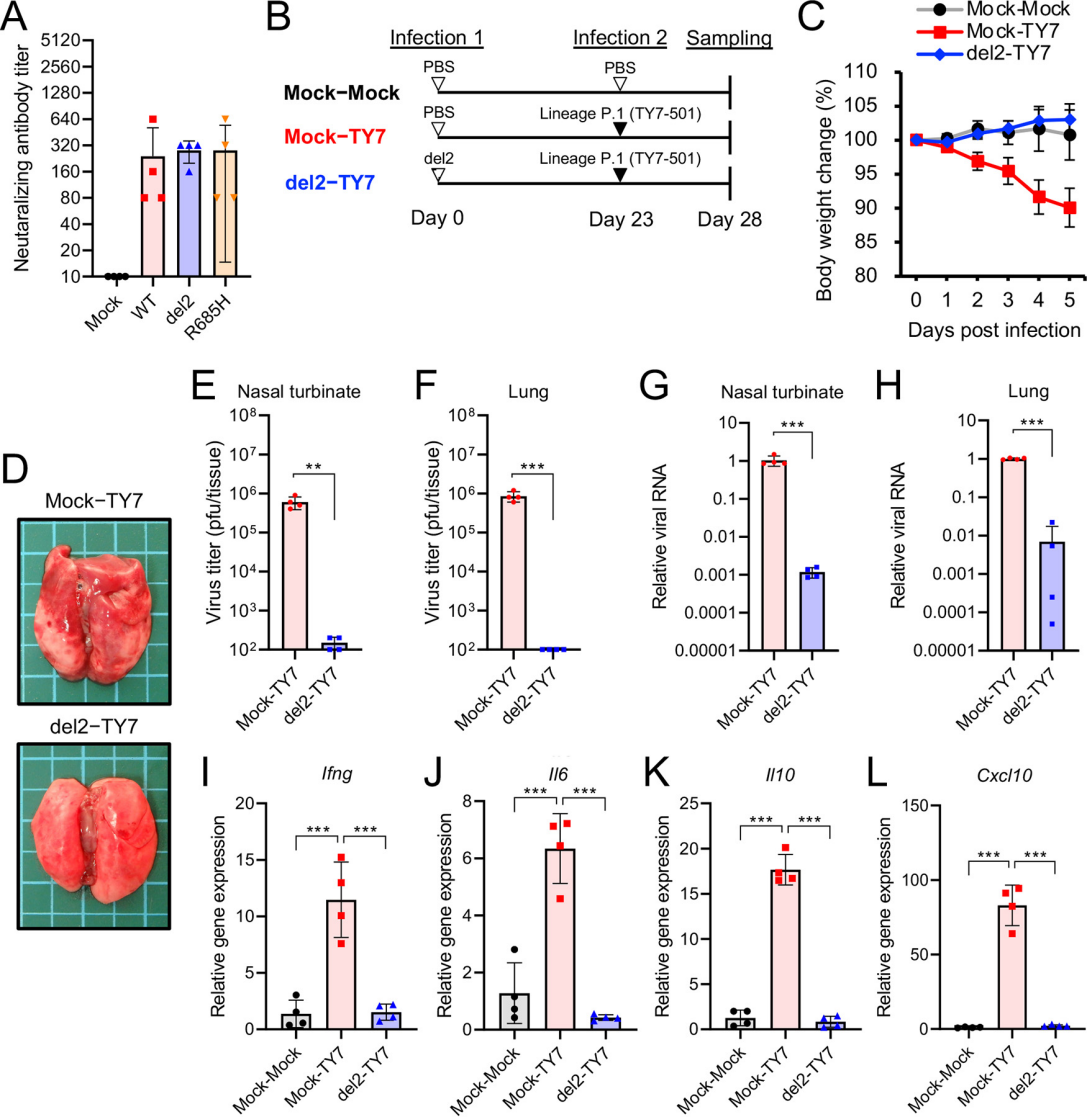
Cross-reactive neutralization among SARS-CoV-2 lineage A and lineage P.1 in hamsters. (A) Cross-neutralization test using SARS-CoV-2 TY7-501 variant (lineage P.1) and hamster sera at 19 days postinfection (dpi) with WT or S gene mutants of SARS-CoV-2 WK-521 (lineage A). (B) Schematic of primary infection, reinfection, and sampling. Hamsters were intranasally inoculated with 1.5 × 10^4^ PFU of WK-521 del2 mutant or PBS. At 23 days post-primary infection, hamsters were infected with 1.5 × 10^5^ PFU of TY7-501 variant. Mock-infected hamsters (mock-mock) and primary-infected hamsters (mock-TY7) were used as controls. (C) Mean body weight changes of hamsters from 0 to 5 days postreinfection. Sample sizes for all groups was as follows: *n *=* *4. (D) Gross pathological images of the lungs of hamsters at 5 days postreinfection. (E and F) Infectious virus titers in the nasal turbinate (E) and lung (F) tissues of hamsters at 5 days postreinfection. Viral titers in the cultures were determined using plaque assays. (G and H) Viral RNA levels relative to primary-infected hamsters (mock-TY7) in the nasal turbinate (G) and lung (H) of hamsters at 5 days postreinfection. The viral RNA levels were quantified using qRT-PCR and normalized to β-actin expression levels. (I to L) Relative gene expression levels of the indicated cytokines in the lungs compared with the levels in the lungs from mock-infected hamsters (mock-mock) were examined using qRT-PCR. Data were normalized to β-actin expression levels. One-way analysis of variance with Tukey’s test was used to determine the statistical significance of the differences. **, *P < *0.01; ***, *P < *0.001.

In the present study, we used S gene mutants from the SARS-CoV-2 WK-521 strain belonging to lineage A. Recently, SARS-CoV-2 variants belonging to lineages B.1.1.7 (United Kingdom), B.1.351 (South Africa), and P.1 (Brazil) have emerged. These variants possess multiple amino acid mutations in the S protein, resulting in increased transmissibility and altered reactivity against neutralizing antibodies (32–36). We used SARS-CoV-2 strains TY7-501 (lineage P.1) and QK002 (lineage B.1.1.7) to investigate whether neutralizing antibodies induced by the infection of the S gene mutant protect from infection with different SARS-CoV-2 lineages. Hamster sera in the convalescent phase of the infection of WK-521 WT or S gene mutants showed neutralizing activity against both TY7-501 and QK002 variants (Fig. 5A and Fig. S2A), whereas the cross-reactivity observed with TY7-501 was lower than that with QK002, presumably owing to the K417T, E484K, and N501Y substitutions in the S protein of TY7-501 (Fig. S3) (32–36). Further, we examined whether primary infection with the WK-521 del2 mutant protects from secondary infection with TY7-501 and QK002 (Fig. 5B and Fig. S2B). Hamsters infected with del2 mutants developed no body weight loss (del2-TY7 in Fig. 5C and del2-QK002 in Fig. S2C) and no macroscopic changes in the lung at 5 days postreinfection with TY7-501 and QK002 (Fig. 5D and Fig. S2D). In the nasal turbinate and lung tissues of del2-TY7 and del2-QK002-infected hamsters, the virus titers were similar to or below the detection limit of the plaque assay (Fig. 5E and F and Fig. S2E and F). Moreover, the viral RNA levels were decreased by primary infection with the del2 mutant (Fig. 5G and H and Fig. S2G and H). Consistent with the low viral levels, cytokine expression levels in del2-TY7- and del2-QK002-infected hamsters were significantly lower than those in naive SARS-CoV-2 variant-infected hamsters (Mock-TY7 and Mock-QK002) (Fig. 5I to L and Fig. S2I to L). Our results indicated that infection with the S gene mutant del2 elicited cross-reactive immune responses to SARS-CoV-2 variants belonging to distinct lineages.

10.1128/mBio.01415-21.2 FIG S2 Cross-reactive neutralization among SARS-CoV-2 lineage A and lineage B.1.1.7 in hamsters. (A) Cross-neutralization test using SARS-CoV-2 QK002 variant (lineage B.1.1.7) and hamster sera at 19 days postinfection (dpi) with WT or S gene mutants of SARS-CoV-2 WK-521 (lineage A). (B) Schematic representation of primary infection, reinfection, and sampling. Hamsters were intranasally inoculated with 1.5 × 10^4^ pfu of WK-521 del2 mutant or PBS. At 23 days post-primary infection, hamsters were infected with 1.5 × 10^5^ pfu of QK002 variant. Mock-infected hamsters (mock-mock) and primary-infected hamsters (mock-QK002) were used as controls. Mock-mock hamsters were the same individuals as those represented in Fig. 4. (C) Mean body weight changes of hamsters from 0 to 5 days postreinfection. The sample size for all groups was as follows: *n *=* *4. (D) Gross pathological images of the lungs of hamsters at 5 days postreinfection. (E and F) Infectious virus titers in the nasal turbinate (E) and lung (F) tissues of hamsters at 5 days postreinfection. Viral titers in the cultures were determined using plaque assays. (G and H) Viral RNA levels relative to primary-infected hamsters (mock-QK002) in the nasal turbinate (G) and lung (H) tissues of hamsters at 5 days postreinfection. The viral RNA levels were quantified using qRT-PCR and normalized to β-actin expression levels. (I to L) Relative gene expression levels of the indicated cytokines in the lungs compared with the levels in the lungs from mock-infected hamsters (mock-mock) were examined using qRT-PCR. Data were normalized to β-actin. One-way analysis of variance with Tukey’s test was used to determine the statistical significance of the differences. **, *P < *0.01; ***, *P < *0.001. Download FIG S2, TIF file, 0.8 MB. Copyright © 2021 Sasaki et al. 2021 Sasaki et al. https://creativecommons.org/licenses/by/4.0/ This content is distributed under the terms of the Creative Commons Attribution 4.0 International license.

10.1128/mBio.01415-21.3 FIG S3 Multiple amino acid sequence alignment of S protein of SARS-CoV-2. Multiple sequence alignment based on the entire length of the deduced S protein sequence of WK521 (lineage A), QK002 (lineage B.1.1.7), and TY7-501 (lineage P.1.). Amino acid substitutions and deletions in QK002 and TY7-501 are shown as pink and green boxes, respectively. Download FIG S3, TIF file, 1.2 MB. Copyright © 2021 Sasaki et al. 2021 Sasaki et al. https://creativecommons.org/licenses/by/4.0/ This content is distributed under the terms of the Creative Commons Attribution 4.0 International license.

## DISCUSSION

Hamsters are vulnerable to SARS-CoV-2 infection, and they develop pneumonia and show marked body weight loss. In the present study, we experimentally infected hamsters with SARS-CoV-2 clinical strains belonging to different lineages. In contrast to clinical strains, tissue culture-adapted S gene mutants bearing mutations at the S1/S2 cleavage site possessed limited growth capacity in hamsters, with no body weight loss and only slight lung damage, as evidenced by histopathological findings and cytokine gene expression levels. These results indicated the attenuated virulence of S gene mutants in hamsters. Furthermore, other studies have reported that the loss of the furin cleavage motif at the S gene results in attenuation and ablated viral growth in hamsters, ferrets, and human ACE2-transgenic mice compared with the original strain carrying the furin cleavage motif (13, 19, 20, 26). Considering the findings from these studies, the low viral growth rate and subsequent mild inflammatory response in the lung tissue are characteristics of attenuated SARS-CoV-2 variants bearing mutations at the furin cleavage site.

The cellular entry mode of S gene mutants accounts for the low growth capacity of S gene mutants in the respiratory airway of hamsters. In the entry phase of SARS-CoV-2 infection, the S protein is primed by host TMPRSS2 or cathepsin and facilitates membrane fusion. The expression of TMPRSS2 in the respiratory airway impacts on the tropism of SARS-CoV-2 (37–39). We have reported that the cellular entry of S gene mutants is triggered by the cathepsin-dependent endosome pathway and not the TMPRSS2-mediated direct viral fusion at the plasma membrane (18). The direct fusion pathway enables SARS-CoV-2 to achieve rapid cellular entry and escape from the innate immune restriction by interferon (IFN)-induced transmembrane proteins (19, 40, 41). S gene mutants thus exhibit low infectivity in certain cell lines, including human lung-derived Calu-3 cells that permit SARS-CoV-2 entry exclusively via the direct fusion pathway (17–19). The inability of the S gene mutants to use TMPRSS2 for S protein activation presumably hampers efficient viral infection and dissemination in airway epithelial cells. Nevertheless, the present study demonstrated that attenuated infection is sufficient for inducing a protective immunity against SARS-CoV-2 infection in hamsters.

Some attenuated virus strains—including the yellow fever virus 17D strain, measles virus Edmonston strain, poliovirus Sabin strain, and varicella zoster virus Oka strain—induce protective immunity in human recipients; therefore, they have been used as live-attenuated vaccines (42). We have thus demonstrated that the SARS-CoV-2 S gene mutants are attenuated variants and can induce protective immunity in hamsters. Primary infection with S gene mutants inhibited viral growth in both the nasal turbinate and lung tissues of hamsters reinfected with a pathogenic clinical strain of SARS-CoV-2. Because the prophylactic administration of neutralizing IgG failed to inhibit the growth of SARS-CoV-2 in the nasal turbinate tissues, the above finding highlights the benefit of vaccination (43). Moreover, inoculation with the S gene mutant del2 induced protective immunity that cross-reacted with currently emerging SARS-CoV-2 variants belonging to the lineages B.1.1.7 and P.1, which escape neutralization by some monoclonal antibodies owing to K417T, E484K, and/or N501Y mutations at the receptor binding domain (RBD) in the S protein (32–36). Although the pathogenicity of S gene mutants in humans remains to be elucidated, this broad neutralizing activity across different lineages indicates the potential of S gene mutants as immunogens in live-attenuated vaccine candidates.

We have some limitations in our study. First, hamsters were rechallenged with SARS-CoV-2 at relatively short intervals (19 to 23 days after initial infection). The longevity of protective immunity needs to be tested in the future to characterize the potential of S gene mutants as immunogens. Second, we cannot exclude the possibility that the nonspecific immune response may be induced by the initial infection and decreases the growth of SARS-CoV-2 in the rechallenged hamsters. In future studies we will address this by challenging with other nonrelated respiratory viruses. Third, the susceptibility and pathogenicity of S gene mutants in other mammals, including humans, remains to be clarified. In humans, naturally arising SARS-CoV-2 variants that lack the furin cleavage motif have been identified as minor populations of quasispecies in clinical specimens from patients with COVID-19 (44). In primary differentiated human epithelial cells, we have demonstrated the low growth properties of S gene mutants. These observations suggested that SARS-CoV-2 mutants that lack the furin cleavage site can infect the human airway, albeit with low growth properties. Further *in vivo* studies using nonhuman primates would provide additional insights on the implications and pathogenicity of S gene mutants. In conclusion, the findings in the present study demonstrated the potential of developing live-attenuated vaccines for the prevention of SARS-CoV-2 infection.

## MATERIALS AND METHODS

### Cells.

Vero (Vero E6; ATCC, Manassas, VA) and Vero-TMPRSS2 (18) cells were maintained in Dulbecco’s modified Eagle’s medium (DMEM) supplemented with 10% fetal bovine serum (FBS). Differentiated human nasal and bronchial epithelial cells (nasal ECs and bronchial ECs, respectively) in an air-liquid interface (ALI) culture were obtained as MucilAir-nasal and MucilAir-bronchial, and maintained in MucilAir culture medium (all from Epithelix, Geneva, Switzerland). All cells were incubated at 37°C with 5% CO_2_.

### Viruses.

SARS-CoV-2 WK-521 (EPI_ISL_408667), QK002 (EPI_ISL_768526), and TY7-501 (EPI_ISL_833366) strains were provided by Masayuki Saijyo, Masayuki Shimojima, and Mutsuyo Ito (National Institute of Infectious Diseases, Japan); the original stock of these virus strains was prepared by inoculation of Vero-TMPRSS2 cells. S gene mutants of SARS-CoV-2 WK-521, del2, and R685H were cloned using a limiting dilution method and propagated in Vero cells (18). The mutations at the S1/S2 cleavage site were verified by whole-genome sequencing (18).

### Ethics statement.

All of the animal experiments were performed in accordance with the National University Corporation, Hokkaido University Regulations on Animal Experimentation. The protocol was reviewed and approved by the Institutional Animal Care and Use Committee of Hokkaido University (approval no. 20-0060).

### Hamster infection.

For virological and histopathological analyses in a single infection, 4- to 6-week-old male Syrian hamsters (Japan SLC, Shizuoka, Japan) were inoculated intranasally with 1.5 × 10^4^ plaque forming units (PFU) of wild-type WK-521 (WT), del2, or R685H viruses in 200 μl of PBS. The body weights of the infected hamsters were monitored daily. At 2, 4, or 19 dpi, a subset of the infected hamsters were euthanized under deep anesthesia by isoflurane inhalation, and tissue samples (nasal turbinate, lung, and blood) were harvested.

For the reinfection experiments, 4-week-old male Syrian hamsters were inoculated intranasally with 1.5 × 10^4^ PFU of WT, del2, or R685H viruses in 200 μl of PBS or PBS only (mock-infected controls). At 19 or 23 dpi, the hamsters were reinfected with 1.5 × 10^5^ PFU of WT, QK002, or TY7-501 virus strains in 200 μl of PBS. At 5 days postreinfection (24 or 28 days post-initial infection), tissue samples (nasal turbinate and lung) were harvested.

Whole lung and nasal turbinate tissues were homogenized in PBS with TissueRuptor (Qiagen, Hilden, Germany). A part of the homogenate was centrifuged for 2 min at 2,310 × *g* to pellet tissue debris, and the supernatant was subjected to plaque assays using Vero-TMPRSS2 cells for virus titration as previously described (18). The remaining part of the homogenate was mixed with TRIzol LS (Invitrogen; Thermo Fisher Scientific, Waltham, MA) and subjected to RNA extraction with a Direct-zol RNA miniprep kit (Zymo Research, Irvine, CA). For relative quantification of viral RNA and host mRNAs, cDNA was synthesized with SuperScript IV VILO master mix (Invitrogen) and analyzed by qRT-PCR with Probe qPCR mix (TaKaRa, Kusatsu, Japan). Target RNA levels were normalized to hamster β-actin and calculated by the ΔΔ*CT* method. Primers and probes for SARS-CoV-2 N (45), primate β-actin (46), and hamster genes (47) were previously described.

### Histopathology and immunohistochemistry.

Nasal turbinate and lung tissue samples were harvested from hamsters infected with SARS-CoV-2 at 2 or 4 dpi. Tissue samples were fixed in 10% phosphate-buffered formalin, and nasal turbinate was decalcified with 10% EDTA solution (pH 7.0). Tissue samples were then embedded in paraffin. The paraffin blocks were sectioned at 4-μm thickness and mounted on Platinum PRO micro glass slides (Matsunami, Osaka, Japan). For histopathological analysis, slides were stained with hematoxylin and eosin (H&E). For immunohistochemical analysis, slides were heated in citrate buffer for 5 min using a pressure cooker for antigen retrieval and blocking with Block Ace (KAC, Kyoto, Japan), followed by staining with anti-SARS-CoV-2 spike antibody (GTX632604; GeneTex, Hsinchu, Taiwan), anti-SARS-CoV-2 nucleocapsid antibody (GTX635679- GeneTex), anti-CD3 (ab16669; Abcam, Cambridge, UK), antimyeloperoxidase (MPO) (A039829-2; Dako; Agilent, Santa Clara, CA), or anti-Iba1 (019-19741, Fujifilm, Wako, Osaka, Japan). Immunostaining was detected by EnVision system peroxidase-labeled anti-rabbit or anti-mouse immunoglobulin (Dako) and visualized with a Histofine diaminobenzidine substrate kit (Nichirei Biosciences, Tokyo, Japan).

### Infection and growth of SARS-CoV-2 in *in vitro* cell culture.

Human nasal ECs and bronchial ECs in an ALI culture were infected at the apical surface with either WT, del2, or R685H viruses at a multiplicity of infection (MOI) of 0.1. After 1 h of incubation, the apical area of cells was washed three times with PBS, and then cells were maintained under ALI culture conditions. At 24, 48, and 72 h postinfection (hpi), 200 μl of culture medium was added at the apical side, and the fluid was harvested for virus titration after 20 min of incubation. Vero cells were infected with either WT, del2, or R685H viruses at an MOI of 0.01. After 1 h of incubation, cells were washed three times with PBS and then cultured in fresh medium with 2% FBS. The culture supernatants were harvested at 24, 48, and 72 hpi. Virus titers were determined by plaque assays as previously described (18). For viral RNA quantification, RNA was extracted with a Direct-zol RNA miniprep kit at 48 hpi (Vero cells) or 72 hpi (Human nasal ECs and bronchial ECs) and analyzed with qRT-PCR with the Thunderbird Probe one-step probe qRT-PCR kit (Toyobo, Osaka, Japan). Viral RNA levels were normalized to nonhuman primate β-actin or human β-actin (Hs99999903_m1; Applied Biosystems; Thermo Fisher Scientific) and calculated using the ΔΔ*CT* method (46).

### Virus neutralization assays.

Serum samples were collected from hamsters at 19 dpi after infection with the SARS-CoV-2 WK-521 strain and heat-inactivated at 56°C for 30 min. Serial 2-fold dilutions of serum samples in DMEM containing 2% FBS were incubated with 160 PFU of SARS-CoV-2 WK-521, QK002, or TY7-501 strains at 37°C for 1 h. The serum-virus mixtures were then added to Vero-TMPRSS2 cells in 96-well plates. After 4 dpi, viral cytopathic effects were examined under an inverted microscope. The neutralization titer was defined as the reciprocal of the highest serum dilution that completely inhibited the cytopathic effect.

### Statistical analysis.

Data were expressed as the mean ± standard deviation (SD). Statistical analysis was performed using one-way analysis of variance (ANOVA) with Tukey’s test using Prism 8 (GraphPad Software, San Diego, CA).
