# Supplementary figures and images for: SARS-CoV-2 Bearing a Mutation at the S1/S2 Cleavage Site Exhibits Attenuated Virulence and Confers Protective Immunity
Source: mBio. 2021 Aug 24;12(4):e01415-21. doi: 10.1128/mBio.01415-21 (PMC8406294; doi:10.1128/mBio.01415-21)

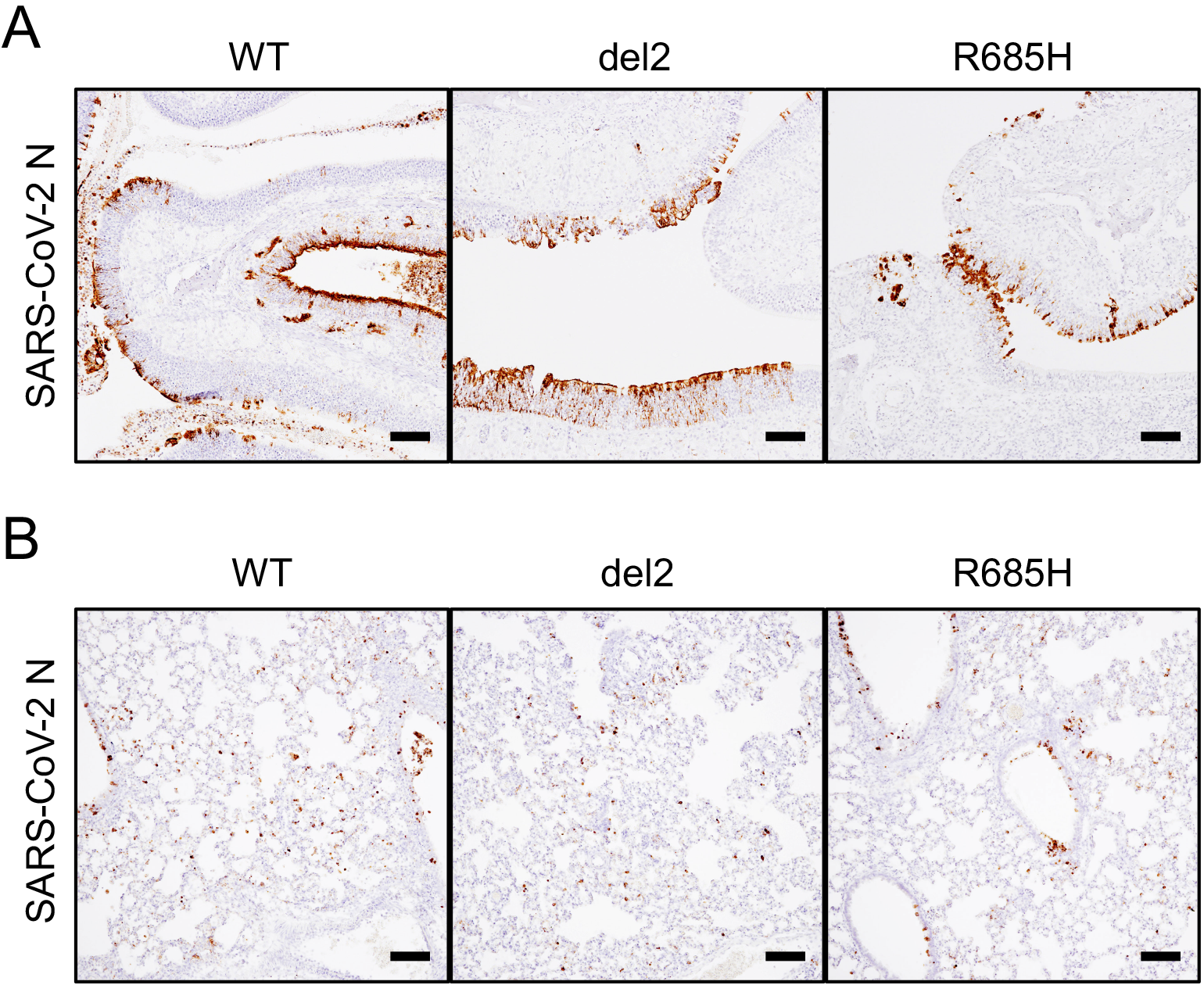

Supplement: FIG S1 [file mbio.01415-21-sf001.tif]

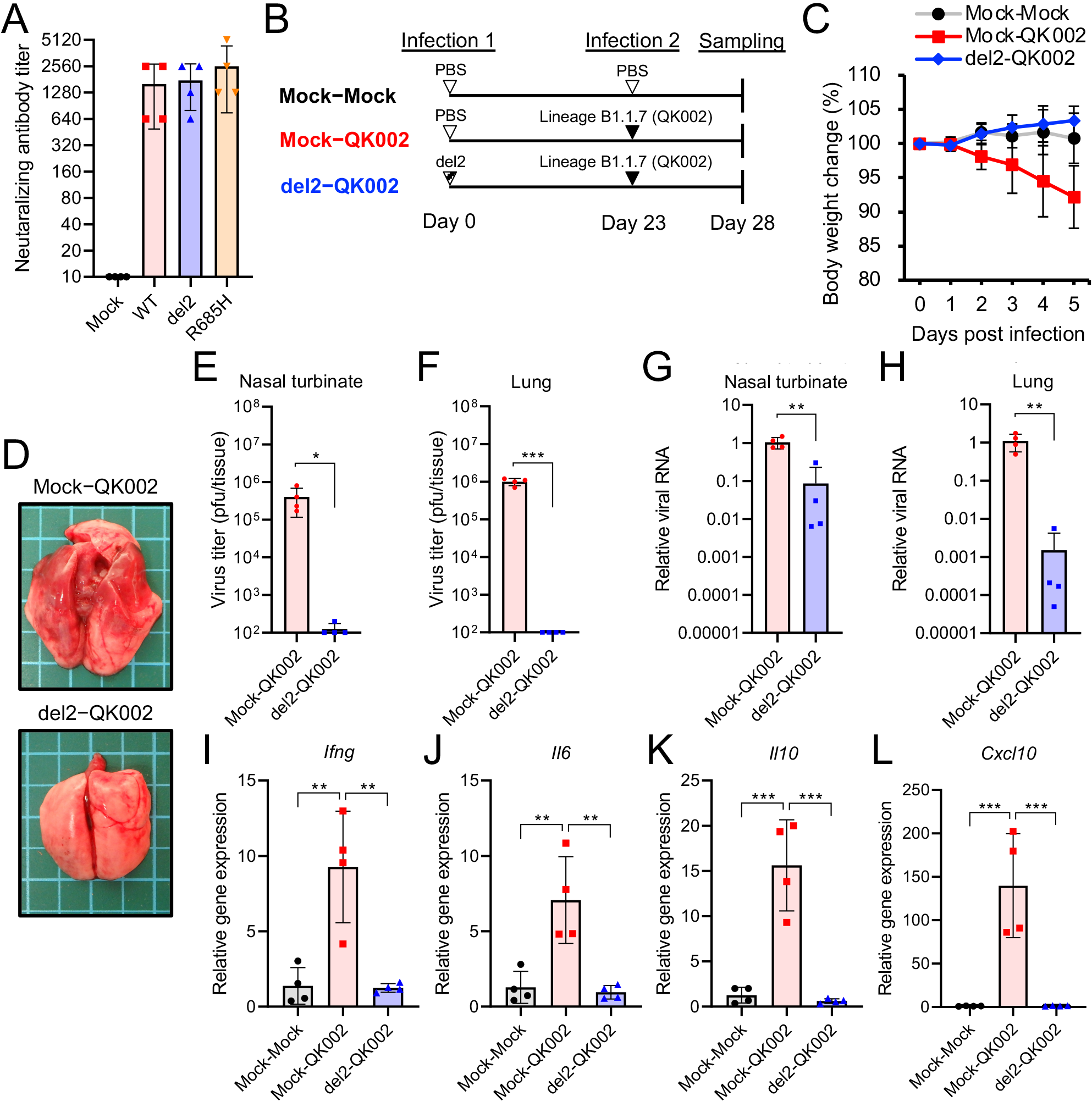

Supplement: FIG S2 [file mbio.01415-21-sf002.tif]

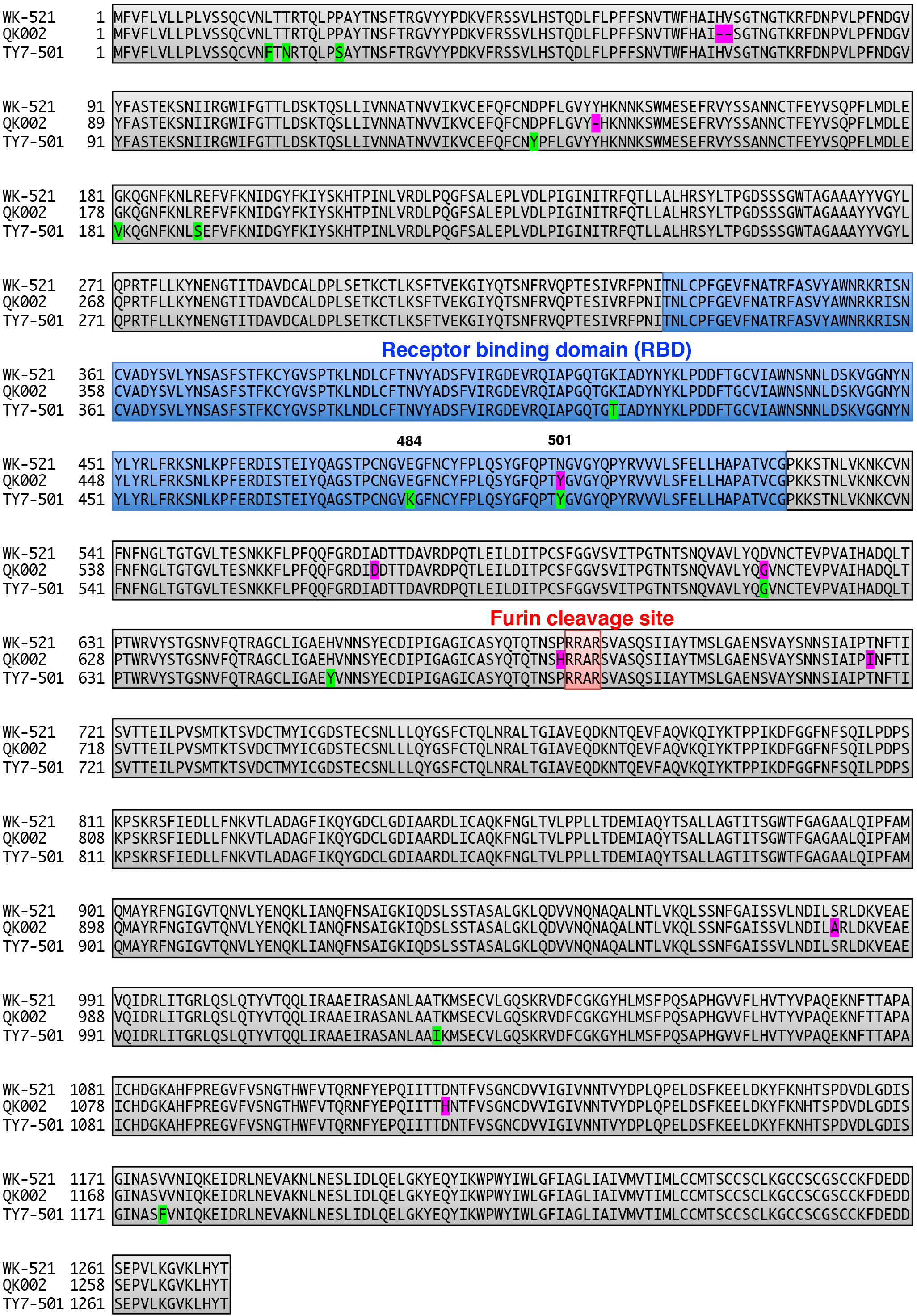

Supplement: FIG S3 [file mbio.01415-21-sf003.tif]
